# The Effects of Multidisciplinary Intensive Rehabilitation on Cognitive and Executive Functions in Parkinson’s Disease: A Clinical Database Analysis

**DOI:** 10.3390/jcm13133884

**Published:** 2024-07-02

**Authors:** Ivana Baldassarre, Rossella Rotondo, Laura Piccardi, Lorenza Leonardi, Danilo Lanni, Maria Gaglione, Fabrizio Stocchi, Massimo Fini, Michela Goffredo, Elvira Padua, Maria Francesca De Pandis

**Affiliations:** 1 San Raffaele Cassino, 03043 Cassino, Italy; ivana.baldassarre@sanraffaele.it (I.B.); rossella.rotondo@sanraffaele.it (R.R.); laura.piccardi@uniroma1.it (L.P.); lorenza.leonardi@sanraffaele.it (L.L.); danilo.lanni@sanraffaele.it (D.L.); maria.gaglione@sanraffaele.it (M.G.); 2 Department of Psychology, Sapienza University of Rome, 00185 Rome, Italy; 3 IRCCS San Raffaele Roma, 00163 Rome, Italy; fabrizio.stocchi@sanraffaele.it (F.S.); massimo.fini@sanraffaele.it (M.F.); michela.goffredo@sanraffaele.it (M.G.); 4 Department of Human Sciences and Promotion of the Quality of Life, San Raffaele Open University, 00166 Rome, Italy; elvira.padua@uniroma5.it

**Keywords:** physical activity, cognitive functions, executive functions, Parkinson’s disease, multidisciplinary rehabilitation, clinical practice

## Abstract

**Background/Objectives:** This study is based on data collected from a medical health record review to assess whether multidisciplinary intensive rehabilitation treatment in Parkinson’s disease (PD) patients can improve global cognitive functioning and executive functions. **Methods**: The data related to PD patients were extrapolated from a clinical database called “NeuroRehab”. A total of 104 PD patients (51 males; 53 females) performed 6 weeks of multidisciplinary intensive rehabilitation treatment in clinical practice from January 2019 to May 2023. This training program was characterized by three daily sessions of 60 min of activities (muscle relaxation and stretching exercises, moderate physical aerobic exercise, and occupational therapy). The patients were classified and stratified according to disease severity (according to the Hoehn and Yahr scale), postural instability and gait difficulty (PIGD) or tremor-dominant (TD) subtypes, disease duration (DD), and the presence of dyskinesias. The effect of multidisciplinary intensive rehabilitation treatment on cognitive and executive functions was evaluated through the administration of cognitive tests, such as the Mini–Mental State Examination (MMSE), Montreal Cognitive Assessment (MoCA), and Frontal Assessment Battery (FAB). All the parameters were evaluated at the baseline (T0) and at the end of the rehabilitation program (T1). **Results**: The multidisciplinary intensive rehabilitation treatment significantly improved cognitive performance. The MMSE, MoCA, and FAB test scores after the rehabilitation program (T1) were significantly higher compared to the scores obtained at the baseline (T0). Moreover, further analyses on subgroups of the patients who scored below the cut-off in the MMSE showed that at least 50% of patients overcame the cut-off score. Interestingly, the same analyses performed for the MoCA and FAB revealed a higher rate of improvement in cognitive functions, with normal scores in both tests after 6 weeks of multidisciplinary intensive rehabilitation treatment. **Conclusions**: This study revealed the potential effects of a 6-week multidisciplinary rehabilitation program in improving cognitive status in a PD inpatient cohort.

## 1. Introduction

Chronic conditions and diseases significantly impact society, leading to disability, health-related retirement, and even premature death. The economic and social costs of these issues are considerable. In the US, between 2016 and 2017, neurodegenerative diseases affected 4.7 to 6.0 million people and were the cause of 272,644 deaths [1,2,3,4,5]. By 2050, the number of people with dementia is expected to increase to 152.8 million, much higher than the 57.4 million recorded in 2019. The estimates suggest that the number of people affected ranges from 50.4 to 65.1 million and from 130.8 to 175.9 million, emphasizing the importance of this issue [6]. In particular, among neurodegenerative diseases, Parkinson’s disease (PD) affects many people, and the number of affected individuals is expected to increase by 2040, reaching over 17 million. Given this projection, it is crucial to prioritize the health, social, and economic impacts of this debilitating condition [7].

PD is characterized by motor symptoms, such as bradykinesia, rigidity, postural instability, and resting tremor, and by non-motor symptoms, including cognitive impairments and depression, that negatively influence the quality of life (QoL) of PD patients [8,9,10,11].

Within the motor symptoms, two distinct PD phenotypes have been identified, commonly referred to as postural instability and gait difficulty (PIGD) and tremor-dominant (TD) subtypes [11]. The PIGD phenotype displays bradykinesia and rigidity, whereas the tremor-dominant (TD) phenotype is characterized by resting tremor, normal gait, and mild progression of the disease [12]. Compared with the TD patients, the patients with the PIGD phenotype experience greater subjective intellectual, motor, and occupational impairments [11]. Moreover, within PIGD, a higher incidence of dementia [13] and neuropsychiatric disorders, such as depression [14] and apathy [15], was reported.

Despite the intense research in the pharmacological and surgical fields, the disease, especially within the PIGD subtype [11], progresses, and PD patients experience severe disability [16].

Following the Committee of Ministers’ Recommendation No. R (92) 6 to Member States of the Council of Europe on a coherent policy for people with disabilities (adopted by the Committee of Ministers on 9 April 1992), the Italian Ministry of Health introduced the Individual Rehabilitation Project (IRP) and published the guidelines for rehabilitation to define the methods and procedures to be provided to assure different settings of rehabilitation, such as an extensive outpatient rehabilitation program and an intensive hospitalization program (Italian Ministry of Health, 1998).

Intensive treatment has proven to be effective for both the motor and non-motor symptoms of PD through various studies [17,18,19,20,21]. For example, it has been shown that a multidisciplinary rehabilitation program (physical therapy, occupational therapy, and speech therapy) significatively improved cognitive performance in the early–moderate PD stages [20], and it appears to improve the QoL in patients with PD in both the short-term and long-term [17,18,20]. Moreover, the positive effects of an intensive multidisciplinary program are apparent in PD patients in the advanced stages of the disease [19].

A broad spectrum of findings about the effects of physical exercise programs on cognitive functions in PD patients is controversial. In a systematic review on PD, da Silva and collaborators [22] highlighted that physical exercise training improves the performance of global cognitive functions. In contrast, a recent meta-analysis did not find any significant result of physical exercise programs on global cognitive function in patients with PD [23]. However, a more recent meta-analysis [24] suggests that physical exercise interventions are effective in improving global cognitive function and executive function in PD patients. The results highlighted that both short-term (ranging between 4 and 12 weeks) and long-term (>12 weeks) treatments, with at least 60 min daily of combined exercise programs, positively affect global cognitive functions. Nonetheless, it is relevant to note that the specificity of physical exercise prescription in PD patients in terms of the frequency, intensity, duration, and type to maximize its beneficial effects on global cognitive functions is still unclear [24]. In this regard, a broad spectrum of cognitive dysfunction has been recognized in PD patients, ranging from mild cognitive impairment (PD-MCI) to PD dementia (PD-D) [25]. Mild cognitive impairment (MCI) represents an intermediate cognitive stage between normal cognitive functioning and dementia. It is associated with a six times higher risk of developing dementia in PD patients than in the general population, and the risk is most significant in those presenting with PD-MCI [26]. Baiano and collaborators [26] found that around 40% of people with PD had mild cognitive impairment (MCI). Furthermore, they identified the most frequently observed phenotype of PD-MCI was the multiple-domain subtype, which means deficits in more cognitive processes than just the memory. It accounted for around 31% of cases. A recent meta-analysis showed that, within three years, 25% of people with Parkinson’s disease and normal thinking abilities developed mild cognitive impairment (PD-MCI), while 2% developed dementia (PD-D). Among those with PD-MCI, 20% developed dementia, while 28% returned to having normal thinking abilities. Moreover, the conversion rates to MCI and dementia were higher and the reversion rates were lower when the follow-up was ≥3 years [27].

The main aim of this study is to investigate whether the Italian multidisciplinary intensive rehabilitation treatment for PD patients leads to beneficial effects on global cognition and executive functions in real-world clinical practice, using the data from retrospective analyses of a medical database.

## 2. Materials and Methods

### 2.1. Study Design

We conducted a study on a pre-existing database, which included data from PD patients collected during a real-world inpatient clinical rehabilitation setting. In order to assess the impact of multidisciplinary intensive rehabilitation on global cognitive performances, a dedicated database, called “NeuroRehab”, was created. NeuroRehab was specifically designed to select the data of PD inpatients who underwent a comprehensive multidisciplinary intensive rehabilitation treatment according to the Italian Rehabilitation Plan (Italian Ministry of Health, 1998). The medical health records of the patients hospitalized in the Neuromotor Rehabilitation Operative Unit of San Raffaele Cassino Hospital were reviewed.

The data on PD patients’ characteristics, including age, gender, disease duration, and stage of disease, which were evaluated according to the Hoehn and Yahr (H&Y) scale in both ON and OFF medication states; the levodopa daily dose (LEDD); the motor phenotype (PIGD and TD); the motor complications (motor fluctuations, dyskinesia, and freezing of gait); and the tests to assess the global cognitive functions using the Level I Movement Disorder Society (MDS) criteria [25] were all retrieved for all included patients from January 2019 to May 2023.

### 2.2. Participants

The inclusion criteria were as follows: (i) to be diagnosed with PD by a neurologist according to the UK Brain Bank criteria without any other significant neurological or orthopedic problems that would limited the execution of rehabilitation treatment; (ii) to have experienced motor fluctuations; (iii) to have been under stable medication during the training period; and (iv) to have completed a rehabilitation period of hospitalization for at least 6 weeks.

The exclusion criteria were as follows: (i) PD patients who had been hospitalized for orthopedic injuries that required a different rehabilitation program; and (ii) patients who obtained the maximum score in all the tests before starting the multidisciplinary intensive rehabilitation program (T0).

The cognitive tests were as follows:

- Mini–Mental State Examination—MMSE [28]: This is a test consisting of 11 questions aiming to check for cognitive impairment. The maximum score is 30 and a score below 24 indicates a possible cognitive impairment.
- Montreal Cognitive Assessment—MoCA [29]: This is a highly sensitive tool for the early detection of mild cognitive impairment (MCI). The maximum score is 30 and a score below 17.54 indicates a possible cognitive impairment.
- Frontal Assessment Battery—FAB [30]: This is a short screening test for evaluating executive cognitive functions. The maximum score is 30 and a score below 13.5 indicates an executive function impairment.

All PD inpatients were re-assessed by the same tests at the end of the rehabilitation program (T1). The patient’s medical records were carefully reviewed to collect the necessary information.

All the patients admitted to San Raffaele Cassino Hospital signed informed consent forms during the hospitalization.

### 2.3. Clinical Rehabilitation

Following the Italian Rehabilitation Plan guidelines, all PD inpatients performed a multidisciplinary engagement with evaluative and task-oriented treatments for the proper management of the disease. The multidisciplinary training consisted of 6 weeks of three daily sessions of 60 min, 7 days a week. Each patient was trained daily at the same time to minimize the on–off fluctuations and the subsequent response to physical therapy.

The first 1 h session included full-body muscle relaxation and stretching exercises followed by exercises to improve flexibility and exercises for balance and gait on a stabilometric platform with visual cues.

The second session included moderate physical aerobic exercise consisting of 45 min treadmill training, setting the treadmill parameters at the maximum tolerated walking speed determined before the training session. The treadmill speed was incremented gradually every 3 days. The heart rate and blood pressure were measured before and after each session. The results were recorded for each session. Throughout the treatment, a therapist supervised the patients, standing alongside them. Each session included a 10 min warm-up and a 5 min cool-down. The third 1 h session focused on occupational therapy to improve autonomy in daily living activities.

The training was personalized for each subject involved in this study, based on their individual characteristics, while ensuring an equivalent workload. The treatment included task-oriented exercises, action–observation therapy, and cueing strategies to improve motor skills learning.

### 2.4. Outcome

This study aims to assess the impact of a 6-week multidisciplinary intensive rehabilitation treatment on PD patients in clinical practice. It will evaluate the following:

- The effect of multidisciplinary intensive rehabilitation on cognitive and executive functions through the analysis of the pre- and post-scores obtained in the MMSE, MoCA, and FAB tests.
- The frequency of patients whose performances at cognitive tests at the end of the rehabilitation treatment switch from a critical score to a normal one.
- The potential effects of other variables, such as motor phenotype, disease duration, the presence of dyskinesias, and H&Y score, on cognitive and executive functions.

### 2.5. Statistical Analysis

The data were entered into SPSS to compute descriptive statistics. A series of repeated measures ANCOVA was performed with the pre- and post-scores of the MMSE, MoCA, and FAB tests as the within-subjects variables and the H&Y scale, motor phenotype (TD vs. PIGD), DD (disease duration: 0–5; 6–10; 11–15; 16–20; and >20) and dyskinesia (presence vs. absence) as the between-subjects variables. Age and gender were included in the models as covariates. Successively, we recoded the new variables of MMSE, MoCA, and FAB, differentiating patients who scored below or above the cut-off, and we performed further analysis in order to investigate a possible switch from abnormal to normal scores related to the rehabilitation program. For all the analyses performed, a *p*-value less than 0.05 was considered statistically significant.

## 3. Results

### 3.1. Data Collection

**Figure 1:**
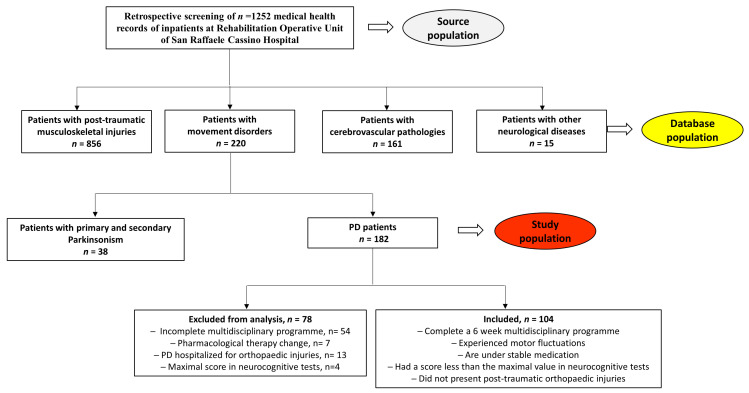
NeuroRehab database. A total of 1252 patients were classified into four groups: patients with post-traumatic musculoskeletal injuries (*n* = 856), cerebrovascular pathologies (*n* = 161), other neurological diseases (*n* = 15), and movement disorders (*n* = 220). Among the movement disorders, the patients were divided into primary and secondary Parkinsonism (*n* = 38) and PD patients (*n* = 182). The PD patients who did not complete a 6-week intense multidisciplinary rehabilitation program (*n* = 54), who were hospitalized for orthopedic injuries (*n* = 13), who changed their pharmacological therapy during the training period (*n* = 7), and who exhibited the maximal score at the baseline in the cognitive tests (*n* = 4) were excluded from the analysis. In grey source population of total inpatient; in yellow database population with subgroups; in red the study PD population.

A retrospective screening of the medical health records of patients hospitalized in the Neuromotor Rehabilitation Operative Unit of San Raffaele Cassino Hospital was performed. A total of 1252 medical health records were consulted, and the patients were classified as patients with post-traumatic musculoskeletal injuries (*n* = 856), cerebrovascular pathologies (*n* = 161), movement disorders (*n* = 220), and other neurological diseases (*n* = 15). Among the movement disorders, the patients were divided into primary and secondary Parkinsonism (*n* = 38) and PD patients (*n* = 182). The PD patients who did not complete a 6-week intense multidisciplinary rehabilitation program (*n* = 54), who were hospitalized for orthopedic injuries (*n* = 13), who changed their pharmacological therapy during the training period (*n* = 7), or who exhibited the maximal score at the baseline in the cognitive tests (*n* = 4) were excluded from the analysis (Figure 1).

### 3.2. Participants

The data included a total of 104 inpatients collected from January 2019 to May 2023 who underwent rehabilitation treatment at San Raffaele Cassino Hospital. Their medical records were reviewed and collocated in the NeuroRehab database. The baseline patients’ characteristics are summarized in Table 1. All the PD patients included in the database experienced motor fluctuations and were under stable medications during the rehabilitation treatment. The median age of the hospitalized PD patients was 72.3 ± 8.7 years, with a prevalence of females. The average disease duration was 13.0 ± 5.9 years, which correlates with a disease progression as indicated by a median H&Y score of 3.6 ± 0.5 in an OFF medication condition. According to the literature [11,31], a higher prevalence of PD inpatients had a PIGD phenotype (61.5%), rather than the TD subtype (38.5%). In contrast to the literature [32], a similar prevalence among the PD-MCI group can be appreciated among the PIGD and TD phenotypes (respectively, 15% vs. 9.4% for the MoCA evaluation and 22.5% vs. 14.1% for the FAB assessment). This feature may be attributed to a faster rate of cognitive decline among the PIGD patients, which makes these patients hospitalized in a neuromotor rehabilitation unit non-cooperative with respect to performing rehabilitation.

### 3.3. Data Analysis

The baseline (T0) and post-treatment (T1) mean and standard deviation (SD) of the outcome measures are reported in Table 2. The data were first analyzed considering all the PD patients. The results, reported in Table 2, showed the main effects of the multidisciplinary intense rehabilitation on cognitive performances: the PD patients’ score on cognitive tests were significantly higher post-treatment (T1) than at the baseline (T0). No interaction effect emerged due to disease duration, motor phenotype, dyskinesias, or the H&Y score for the MMSE and FAB tests. As regards the MoCA analyses, an interaction effect “MoCA scores × Dyskinesias” emerged (F = 6.258; *p* < 0.05; and *p*-η^2^ = 0.06). A post hoc analysis revealed that the MoCA scores at the baseline were significatively higher in the PD patients with dyskinesias compared to the PD patients who did not experience dyskinesias (M = 24.12 vs. M = 22.03), whereas no difference between the groups was observed in the MoCA scores at the end of the treatment (T1).

**Figure 2:**
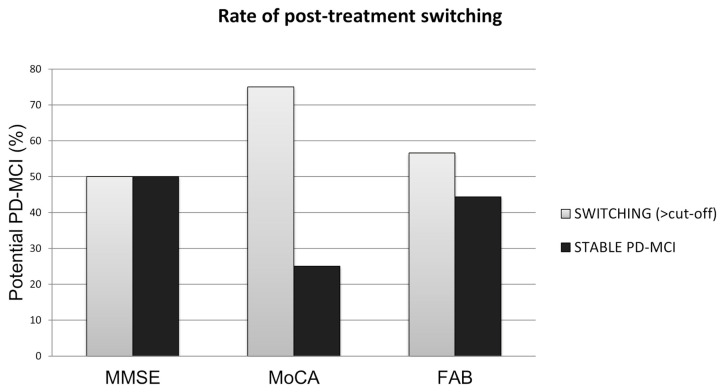
Comparison of the percentages of possible PD-MCI patients whose cognitive performances improved, highlighting the switch from an altered to a normal score, after the multidisciplinary intensive rehabilitation program.

Regarding the MMSE, a consistent group of patients scored below 24, indicating possible cognitive impairment (MCI). Therefore, a statistical analysis of a cohort of PD patients with potential MCI (*n* = 18) and without MCI (*n* = 86) was conducted. Within a 6-week multidisciplinary rehabilitation program, in those with a possible PD-MCI, 50% (nine participants) switched to a normal score (Figure 2), and 50% (nine participants) remained under stable MCI at the end of the rehabilitation program (T1). Moreover, the MMSE increase remains statistically significant, excluding the PD-MCI patients and considering only the PD patients with scores higher than 24 (Table 3).

Interestingly, when the same analyses were performed for the MoCA and FAB, considering a cut-off of 17.54 and 13.5, respectively, it can be observed that a multidisciplinary intensive rehabilitation program led to a higher rate of reversion back to normal scores in both tests. To a lesser extent, the benefits of the multidisciplinary intensive rehabilitation treatment were also revealed in the results of the FAB assessment, since 55.6% (ten participants) of those patients with MCI reverted back to PD-N, and a general trend toward a positive increase in cognitive functions was visible in the 44.4% (eight participants) remaining possible PD-MCI patients. Finally, similar to the MMSE, the exclusion of values less than the reported cut-offs (MoCA < 17.54 and FAB < 13.5) did not influence the statistical significance of the results in the general PD population analyzed (MoCA, *n* = 92 and *p*-value < 0.0001; FAB, *n* = 86 and *p*-value < 0.0001).

## 4. Discussion

Cognitive decline in PD is quite common and heterogeneous [25] and may not be clinically evident, although it is detectable by specific cognitive tests [33]. We compared the performance pre- and post-rehabilitation in three cognitive tests: the MMSE [28], MoCA [29], and FAB [30]. Significant improvements were recorded at the end of the treatment program in all conditions. Our findings indicates that a multidisciplinary intensive rehabilitation treatment can potentially decrease the risk of cognitive decline, helping in ameliorating cognitive performances. This result seems to not be affected by other PD clinical characteristics, such as motor phenotype, disease duration, disease severity, and dyskinesias, in accordance with previous findings suggesting that physical exercise interventions are effective in improving the global cognitive and executive function in PD patients [22,24].

Additional results showed that, at the end of the rehabilitation program, a considerable number of performances in the FAB and MoCA tests obtained a score that exceeded the threshold limit values, which were below the normal cut-offs at the baseline assessment. Regarding the MMSE, despite a significant improvement in the performances recorded at the end of the rehabilitation program, the same trend of switching from an impaired score to a normal one was not observed. This result can be explained considering that several studies highlighted that the MoCA is more accurate than the MMSE in the detection of cognitive impairment as the MMSE showed a lower sensitivity among multiple study settings [34,35,36] and presented ceiling effect problems [37].

Moreover, the MoCA is likely superior to the MMSE for the PD population due to its increased sensitivity in testing the executive abilities commonly affected by PD [38]. As regards the FAB test, previous studies confirm that it is a sensitive tool for detecting dysexecutive functions in the PD population [39,40]. On the basis of these considerations, our study revealed that multidisciplinary rehabilitation improves executive functions in PD patients, positively influencing the reversion to a normal cognitive FAB score. However, this observation seems to not be influenced by motor phenotype as previously reported in the literature [11,31]. The explanation must be sought in the small number of PD-MCI within the PIGD phenotype, which is not representative of this PD cohort since rapid cognitive decline makes these patients unsuitable for hospitalization in a neuromotor rehabilitation unit.

The research into the effectiveness of physical therapies for Parkinson’s disease is essential and appreciated since there is no agreement on the most suitable intensity, frequency, or duration of these therapies. One example of such research is the systematic review conducted by Rotondo et al., 2023 [41], demonstrating the need for more studies on this topic. Moreover, these studies aid in identifying the protective factors that can decrease the likelihood of individuals with mild cognitive impairment and Parkinsonism developing Parkinson’s dementia. Understanding which intervention can reduce this risk and has cognitive benefits fundamentally impacts the quality of life of these patients and their caregivers. Anyone with a long-term neurodegenerative disease knows how crucial it is to maintain a high quality of life as long as possible.

Although our study obtained promising results, it is important to acknowledge certain limitations. Firstly, the nature of the retrospective study on a pre-existing database excludes the possibility of having a control group or assessing the impact of other measurements not included in the database (for example, MDS-UPDRS, depression scales, etc.).

Worth noting is that the influence of learning effects in some items of administrated cognitive tests cannot be excluded. This well-known phenomenon in clinical neuropsychology may at least partly explain our results. In order to avoid this primacy effect, alternative versions of the cognitive tests, whenever possible, might be considered for future research.

Furthermore, this study was a short-term study and did not include a clinical follow-up to determine the longevity of the cognitive benefits. Moreover, during the period considered, the COVID-19 pandemic interrupted the continuity of care for patients with chronic conditions; therefore, the number of participants who fell below the cut-off point was too small to fully rely on the results, highlighting the need for more data.

Since the current estimates project the number of individuals with PD to be over 17 million by 2040 [7], strategies for dementia risk reduction in PD-MCI cases and prevention deserve particular attention. In this regard, additional randomized controlled studies are necessary to explore and better understand the most effective multimodal rehabilitation program able to induce a disease-modifying treatment for cognition in the PD population.

## Figures and Tables

**Table 1:** PD patients’ characteristics at baseline.

**Gender**	***n* (%)**
Male	51 (49.0)
Female	53 (51.0)
Total PD patients	104 (100)
**Age**	***n* (%)**
<60 years	10 (9.6)
60–69 years	25 (24.0)
70–79 years	48 (46.2)
>80 years	21 (20.2)
Mean ± SD	72.3 ± 8.7
**Education levels**	
0–3 years	1 (1)
4–5 years	50 (48.1)
6–8 years	30 (28.8)
9–13 years	20 (19.2)
>13 years	3 (2.9)
Mean ± SD	7.8 ± 3.5
**Disease Duration**	***n* (%)**
<6 years	6 (5.8)
6–10 years	33 (31.7)
11–15 years	36 (34.6)
16–20 years	20 (19.2)
>20 years	9 (8.7)
Mean ± SD	13.0 ± 5.9
**H&Y stage (OFF medication)**	***n* (%)**
2 = Bilateral involvement without impairment of balance	0 (0.0)
2.5 = Mild bilateral disease with recovery on pull test	2.5 (0.0)
3 = Mild to moderate bilateral disease; some postural instability; physically independent	43 (41.3)
4 = Severe disability; still able to walk or stand unassisted	61 (58.7)
Mean ± SD	3.6 ± 0.5
**H&Y stage (ON medication)**	***n* (%)**
2 = Bilateral involvement without impairment of balance	9 (8.7)
2.5 = Mild bilateral disease with recovery on pull test	39 (37.5)
3 = Mild to moderate bilateral disease; some postural instability; physically independent	53(51.0)
4 = Severe disability; still able to walk or stand unassisted	3 (2.9)
Mean ± SD	2.8 ± 0.4
**LEDD (mg), Mean ± SD**	943.5 ± 360.7
**MOTOR PHENOTYPE**	***n* (%)**
Tremor-dominant	40 (38.5)
Akinetic–rigid subtype	64 (61.5)
**MOTOR COMPLICATIONS**	***n* (%)**
Motor fluctuations	104 (100)
Dyskinesia	71 (68.3)
FOG	21 (20.2)

**Table 2:** The significant results of the repeated measures ANCOVA analyses with the pre- and post-treatment scores of the MMSE, MoCA, and FAB tests as the within-subjects variables and the H&Y scale, motor phenotype (TD vs. PIGD), DD (disease duration: 0–5; 6–10; 11–15; 16–20; and >20), and dyskinesia (presence vs. absence) as te between-subjects variables. Age and gender were included in the models as covariates.

Test	Pre-Treatment (T0) (*n* = 104)	Post-Treatment (T1) (*n* = 104)	F	*p*-Value	Partial-η^2^
MMSE	25.95	26.99	67.886	*p* < 0.001	0.397
MoCA	22.69	24.42	116.068	*p* < 0.001	0.530
FAB	15.52	16.32	77.65	*p* < 0.001	0.430
MoCA × Diskinesias	22.03 (+)	24.03 (+)	6.258	*p* < 0.05	0.059
	24.12 (−)	25.27 (−)			

Abbreviations: MMSE, Mini–Mental State Examination; MoCA, Montreal Cognitive Assessment; FAB, Frontal Assessment Battery; (+), presence of dyskinesias; and (−), absence of dyskinesias.

**Table 3:** Assessment of global cognitive functions in the subgroups that scored below or above the cut-off.

Subgroup Analysis	Pre-Treatment (T0)	Post-Treatment (T1)	*p*-Value
MMSE < 24, *n* = 18	21.1 ± 2.7	22.9 ± 3.1	*p* < 0.001
MMSE ≥ 24, *n* = 86	27.0 ± 1.8	27.8 ± 1.5	*p* < 0.001
MoCA < 17.54, *n* = 12	15.0 ± 1.6	18.6 ± 2.0	*p* < 0.001
MoCA ≥ 17.54. *n* = 92	23.7 ± 2.8	25.2 ± 2.3	*p* < 0.001
FAB < 13.5, *n* = 18	12.1 ± 1.2	13.7 ± 1.3	*p* < 0.001
FAB ≥ 13.5, *n* = 86	16.2 ± 1.4	16.9 ± 1.0	*p* < 0.001

Abbreviations: MMSE, Mini–Mental State Examination; MoCA, Montreal Cognitive Assessment; FAB, Frontal Assessment Battery. Paired Student’s t-test with a *p*-value < 0.05 was statistically significant.

## Data Availability

The dataset is available on request from the authors.
